# Feline Immunodeficiency Virus (FIV) Neutralization: A Review

**DOI:** 10.3390/v3101870

**Published:** 2011-10-13

**Authors:** Margaret J. Hosie, Daniela Pajek, Ayman Samman, Brian J. Willett

**Affiliations:** Medical Research Council, University of Glasgow Centre for Virus Research, Henry Wellcome Building for Comparative Medical Sciences, 464 Bearsden Road, Glasgow G61 1QH, UK; E-Mails: d.pajek.1@research.gla.ac.uk (D.P.); aymansamman@gmail.com (A.S.); brian.willett@glasgow.ac.uk (B.J.W.)

**Keywords:** FIV, neutralization, neutralizing antibody, feline immunodeficiency virus

## Abstract

One of the major obstacles that must be overcome in the design of effective lentiviral vaccines is the ability of lentiviruses to evolve in order to escape from neutralizing antibodies. The primary target for neutralizing antibodies is the highly variable viral envelope glycoprotein (Env), a glycoprotein that is essential for viral entry and comprises both variable and conserved regions. As a result of the complex trimeric nature of Env, there is steric hindrance of conserved epitopes required for receptor binding so that these are not accessible to antibodies. Instead, the humoral response is targeted towards decoy immunodominant epitopes on variable domains such as the third hypervariable loop (V3) of Env. For feline immunodeficiency virus (FIV), as well as the related human immunodeficiency virus-1 (HIV-1), little is known about the factors that lead to the development of broadly neutralizing antibodies. In cats infected with FIV and patients infected with HIV-1, only rarely are plasma samples found that contain antibodies capable of neutralizing isolates from other clades. In this review we examine the neutralizing response to FIV, comparing and contrasting with the response to HIV. We ask whether broadly neutralizing antibodies are induced by FIV infection and discuss the comparative value of studies of neutralizing antibodies in FIV infection for the development of more effective vaccine strategies against lentiviral infections in general, including HIV-1.

## 1. Introduction

The lentiviral Env is essential for viral entry, facilitating the attachment of the virus to cells through high affinity interactions with its cellular receptor(s). Infection with the primate and feline lentiviruses requires sequential interactions with a primary receptor and a co-receptor to trigger the conformational change in Env that allows fusion of the viral envelope and cellular membrane to proceed. Whereas CD4 is the primary receptor for human immunodeficiency virus (HIV) and simian immunodeficiency virus (SIV) [1–3], CD134 (OX40) is the primary receptor for feline immunodeficiency virus (FIV) [4]. HIV can use a range of seven transmembrane domain G-protein-coupled receptors as co-receptors for entry, the major co-receptors used are CXCR4, CCR5 and (to a lesser extent) CCR3 [5–9]. In contrast, all strains of FIV tested to date use CXCR4 as a co-receptor [10–15] and CCR5 does not function as a co-receptor for FIV. Env exists on the virion as a complex trimeric structure comprising variable and conserved regions. While conserved epitopes that form the binding site for the primary receptor are accessible to the humoral immune response, the co-receptor binding site is largely hidden and is only exposed following a conformational change triggered by engagement of the primary receptor. Moreover, much of the Env is protected from the humoral response by extensive N-linked glycosylation and by immunodominant epitopes on variable loops such as the third hypervariable loop (V3).

The neutralizing response to HIV has been studied extensively and is reviewed elsewhere [16,17]. It is thought that the majority of HIV neutralizing antibodies are strain-specific, targeting determinants that are unique to a particular viral variant and its genetic relatives. Occasionally, neutralizing antibodies are induced that recognize diverse viral strains; viruses of distinct clades and geographic origins and it is these broadly neutralizing antibodies that are the goal for lentiviral vaccine development.

## 2. Measuring Neutralizing Antibody

### 2.1. Assays for HIV Neutralizing Antibodies

In order to quantify the level of neutralizing antibody in a sample of biological fluid, the sample is mixed with a known quantity of virus or viral antigen. The antibodies within the sample are allowed to interact with the virus before adding the mixture to an indicator cell or system in which the ability of the virus to grow or mediate an effect may be quantified. Thus, early HIV neutralization assays mixed infectious virus with serially-diluted sera before plating onto susceptible cells [18,19]. However, preparing titrated stocks of HIV and quantifying infection in susceptible cells proved experimentally demanding and thus techniques were developed in which the HIV Env was “pseudotyped” onto the surface of the rhabdovirus vesicular stomatitis virus (VSV). The benefits of this technique, developed initially for the study of human T-lymphotropic virus (HTLV) [20], were the ability to prepare high titer stocks of VSV (HIV) pseudotypes that, when plated onto HIV-susceptible indicator cells, gave a marked and measurable cytopathic effect or “plaque assay” that was mediated by replication of VSV in the target cell. Such assays yielded valuable insights into the neutralization of HIV by experimentally-induced immune sera and sera from HIV-infected patients. However, as our understanding of the process of viral entry and replication developed, it became apparent that the strains of virus that grew readily in the laboratory in established cell lines were often not representative of primary field strains of virus. For example, adaptation for growth in established cell lines selected for viral variants that utilized CXCR4 efficiently and, in doing so, altered the sensitivity of the virus to neutralizing antibody. Thus, many researchers consider the “gold standard” neutralizing antibody test to be that based upon primary peripheral blood mononuclear cells (PBMC) as the target cell and uncloned primary clinical (field) isolates. In this assay system, PBMC stimulated with phytohemagglutinin (PHA) are cultured with IL-2 before infection in the presence or absence of the test antibodies. Inhibition of viral infection is measured by the detection of either HIV p24 antigen or reverse-transcriptase (RT) activity in the culture fluids. The main limitation of this system is the inherent variability in susceptibility of PBMCs to infection, which affects the reproducibility of the assay system [21]. Similarly, antibodies targeting the RT enzyme itself may affect an RT assay-based read-out. An alternative assay, updating the pseudotype-based approach introduced by Weiss *et al.* in 1985, utilizes pseudotyped viruses that are generated by co-transfection of an env- and rev-deleted HIV backbone, together with the env clone of interest; the resulting pseudotypes are used to infect a transformed cell line expressing the appropriate viral receptors, such as TZMbl, a genetically engineered HeLa cell clone that expresses CD4, CXCR4 and CCR5 and contains a Tat-responsive reporter gene encoding the firefly luciferase enzyme [22,23]. This pseudotype-based assay has proved to be robust and highly reproducible [24,25]. Other advantages of this system include its shorter assay length (2–3 days compared to 4–6 days for the PBMC-based system) and the lack of cell–cell transmission [21]. However, primary cell-based assays and pseudotype virus assays may each reveal distinct patterns of cross-neutralization [26]. A comparison of the neutralization of a panel of HIV-1 strains by the monoclonal antibodies b12 and 4E10 revealed 84% concordance between the primary cell-based assay and the pseudotype virus assay with monoclonal antibody b12 but only 52% concordance between the two assays with 4E10 [27]. Similarly, an analysis of primary isolate neutralization by pooled plasmas revealed significant, bi-directional discordance between the two assay systems [28]. It is possible that the discordance between the two systems stems from the HeLa-derived TZMbl cells expressing supra-physiological levels of CXCR4 and CCR5 and promoting infection via an endocytic route. In comparison, mitogen-stimulated PBMCs express lower levels of CXCR4 and CCR5 and support infection via direct fusion of the viral envelope and plasma membrane. Thus, while the TZMbl-based assay offers a more robust and reproducible system, the relationship between the neutralizing titers and humoral immunity to infection remains unclear.

### 2.2. Assays for FIV Neutralizing Antibodies

Novel isolates of FIV are conventionally isolated in primary cultures of mitogen-stimulated feline PBMC [29], cells which express both CD134 and CXCR4 [4,30]. However the growth and manipulation of IL2-dependent cultures of feline PBMC is a technically demanding process and thus the propagation of primary strains of FIV proved challenging. Some primary isolates contain viral variants that have the ability to replicate in the (CD134-negative) CrFK fibroblast line [29] as well as in PBMC. These viral variants are easy to propagate and form syncytia or “plagues” in the monolayer of infected cells. Early FIV neutralization assays took advantage of these CrFK-adapted viruses to develop a focus reduction assay in CrFK cells and demonstrated the presence of neutralizing antibody in cats that had been naturally or experimentally infected with FIV [31–33]. Moreover, assays with cat antibodies against homologous or heterologous strains indicated the existence of FIV neutralization subtypes [32]. However, process of adaptation of the viruses for growth in CrFK cells selected for variants that were able to infect cells independently of CD134, via a direct interaction with CXCR4. During this process, the viral Env protein acquired mutations in the V3 loop homologue that increased the net charge of the loop. These mutations modulated (enhanced) the sensitivity of the virus to neutralizing antibodies directed against V3. Therefore, while CrFK-based assays proved informative in regard to the antigenic-relatedness of viruses, their significance to *in vivo* neutralization and vaccine protection was limited. Accordingly, PBMC-based neutralization assays were developed, measuring RT activity in the culture fluids to monitor inhibition of infection [34]. Given the variability of PBMC-based methods, we developed a viral pseudotype-based neutralization assay to detect anti-FIV neutralizing antibodies. A series of HIV (FIV) luciferase pseudotypes were prepared by co-transfecting HEK-293T cells with plasmids encoding: (i) an *env*-deleted HIV provirus incorporating a luciferase reporter gene, pNL-Luc-E-R+ (11); and (ii) an FIV *env* gene expressed from the vector VR1012. The resulting HIV (FIV) luciferase pseudotypes were incubated with serial dilutions of each plasma sample in order to permit neutralization of the pseudotype to proceed, prior to the addition of substrate cells and further incubation. Infection with the residual, non-neutralized pseudotype was then quantified by measuring luciferase activity and the percent neutralization was calculated with reference to control wells containing no plasma. Plasmas were classified as strongly neutralizing (≥80%), moderately neutralizing (60–79%), and weakly neutralizing (40–59%) [35].

### 2.3. Optimization of the CLL-CD134-Based Assay for FIV Neutralizing Antibody

FIV infects cells by sequential interactions with the primary receptor CD134 [4] followed by a second interaction with the chemokine CXCR4 [12,13]. Thus, target cells for FIV neutralization assays should express both CD134 and CXCR4. As CD134 expression in the cat is restricted predominantly to activated CD4+ helper T cells, there are relatively few cell lines that may serve as suitable substrates for FIV neutralizing antibody assays. The MYA-1 cell line, an IL-2 dependent CD4+ feline lymphoblastoid cell line [36] expresses both CD134 and CXCR4 and has been utilized previously in FIV neutralization assays [37]. However, CXCR4 expression on MYA-1 cells is low (approximately 10%), growth is slow, they are sensitive to manipulation and they are IL-2-dependent; all of which conspire to limit the utility of this cell line for routine neutralization assays. As an alternative, and to overcome these limitations, the cell line CLL-CD134, derived from a canine chronic lymphocytic leukemia and transduced with feline CD134 was developed [38]. High expression of CD134 (over 99%) was maintained over 40 passages of the CLL-CD134 cell line; the cells are IL-2 independent, easily manipulated and robust with a high growth rate compared to MYA-1. When the susceptibility of MYA-1 and CLL-CD134 to infection with HIV (FIV) pseudotypes bearing diverse FIV Envs are compared, CLL-CD134 display a similar pattern of susceptibility to infection to MYA-1 cells, but achieve significantly higher counts per minute compared with MYA-1 cells, indicating that CLL-CD134 offered an ideal replacement to MYA-1 cells as the substrate for subsequent FIV neutralizing antibody assays.

## 3. FIV Neutralizing Antibodies

### 3.1. Time Course of Neutralizing Antibody Production in FIV Infection

**Figure 1.**
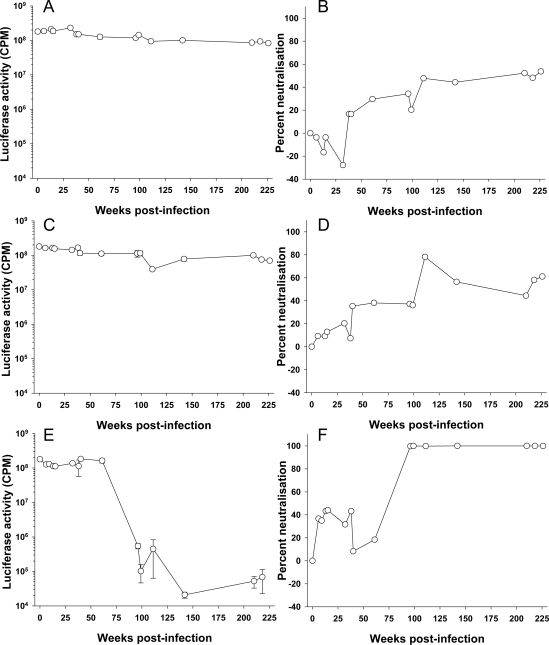
Longitudinal development of virus neutralizing ant ibodies in feline immunodeficiency virus (FIV)-infected cats. Sequential plasma samples collected from three cats (**A**,**B**—611, **C**,**D**—612, **E**,**F**—613) infected experimentally with a molecular clone of FIV GL8 were diluted 1:10 and examined for ability to neutralize HIV (FIV) pseudotypes bearing the GL8 Env as described [35]. Luciferase activity (**A**,**C**,**E**) was measured at three days post-infection and the percentage neutralization (**B**,**D**,**F**) calculated relative to a “no plasma” control. Each point is derived from the mean (n = 3) +/− SEM.

Anti-FIV antibodies can be detected as early as 6 weeks post infection in experimentally infected cats [39]. Similarly, seroconversion can typically be detected between 2 and 4 weeks post infection with HIV [40,41]. However, it takes longer for an anti-HIV-1 neutralizing antibody response to develop and not all infections induce a neutralizing response. Richman *et al.* [42] compared the neutralizing response of three treatment-naive HIV-1 infected individuals to autologous virus and found marked differences in both response time and titer. Neutralizing antibodies capable of neutralizing the homologous virus may develop in the first months of infection [42–44], while cross reactive neutralizing antibodies develop over time in up to 30% of patients [45–48]. Non-neutralizing antibodies against Env may be detected as early as 2 weeks post infection for FIV [49]; however, little is known about the development of neutralizing antibodies in FIV-infected cats. In order to estimate the time required for the development of an anti-FIV neutralizing response in an infected cat, sequential samples collected from three cats infected experimentally with the GL8 molecular clone of FIV were examined for ability to neutralize HIV(FIV) pseudotypes bearing the homologous GL8 Env (Figure 1). The results demonstrate that the neutralizing antibody response to infection develops slowly over time, indeed within the first two years, only weak neutralization was detected (<50% neutralization at a plasma dilution of 1:10). By the end of the study period at approximately four years post-infection, virus neutralization in cats 611 (Figure 1A,B) and 612 (Figure 1C,D) had reached a plateau of 50 to 60%. The humoral response of cat 613 to infection appeared similar to those of 611 and 612 initially, neutralization barely exceeding 40% in the first year. However, by approximately two years (99 weeks), post-infection a sharp increase in neutralizing activity was detected; a potent neutralizing response had been elicited that could reduce luciferase activity >1000-fold. During this period, the proviral load in the three cats remained remarkably stable [50], suggesting that the potent neutralizing response elicited in cat 613 was variant-specific, and did not ultimately influence the proviral burden of the animal. The primary focus for the response was the V5 loop of gp120 and was accompanied by the emergence of neutralization-resistant variants bearing V5 loop mutations (see below and [51,52]). While it is possible that the GL8 strain of FIV may induce neutralizing antibodies very poorly, these preliminary data demonstrate that the neutralizing response to FIV can be both slow to develop and ultimately very weak. Further, given the stark difference between the responses of cats 611 and 612 and that of cat 613, they emphasize that inter-cat variation may be substantial. We have found previously that distinct combinations of viral variants evolved in each of the infected animals (611, 612 and 613) with time post-infection [51]. Accordingly, the nature of the neutralizing antibody response induced in each of the infected cats may have been shaped by both the composition of the viral quasispecies that evolved within the cat (the immunogen) and the ability of the cat to generate a humoral immune response to the virus (the immunogenetics of the host).

### 3.2. Prevalence of Neutralizing Antibodies in FIV Infected Cats

**Figure 2.**
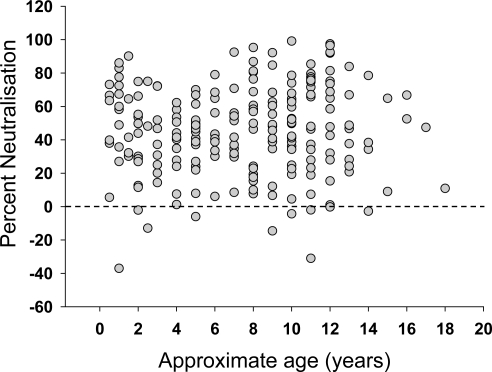
Relationship between estimated age and ability to neutralize FIV. 214 plasma samples from cats testing positive for FIV in the United Kingdom were diluted 1:10 and tested for ability to neutralize GL8 Env-bearing pseudotypes. Graph represents estimated age *versus* percentage neutralization relative to a no plasma control.

Plasma samples were collected from 345 FIV sero-positive, naturally infected, cats across the United Kingdom and submitted to the Companion Animal Diagnostic Service at the University of Glasgow. The cats ranged from 5 months to 18 years of age, although the ages of many of the animals were either unknown or estimated by the cats’ veterinarians. The study population also included FIV-infected cats held in cat protection shelters, strays awaiting adoption, or free-roaming cats. Cats within these groups are at greater risk of FIV infection, according to epidemiological studies [53]. The FIV-infected cats in this study were five times more likely to be male than female, consistent with previous studies which demonstrated that males are at a higher risk of FIV infection due to their more aggressive nature [54]. Approximately one third of the cats displayed no clinical signs and were apparently healthy. However, not all cats had been examined thoroughly by veterinarians and so this proportion may have been overestimated. Clinical signs recorded for the sick cats included weight loss, dullness, anorexia, vomiting, inappetence, malaise, lethargy, pyrexia, anemia, jaundice, keratitis/uveitis, upper respiratory tract signs, stomatitis/gingivitis, neurological signs and enlarged lymph nodes. Samples were screened for neutralizing antibodies using HIV (FIV) pseudotypes bearing the GL8 Env as a representative subtype A UK-derived strain of FIV. Since the plasma samples were collected from clinical cases, the time post-FIV infection was not known. However, comparison of the GL8 neutralizing activity revealed a full spectrum of activities, from no neutralization to 100% neutralization. It has been suggested that the neutralizing response may broaden with time post-infection, indeed, our own data from cats 611, 612 and 613 (Figure 1) suggest a slow, but significant, increase in the response with time post-infection. Therefore, since older FIV-positive cats may have been infected for longer, we examined the data collected from a subgroup of 214 cats for which age had been recorded, to determine whether older cats had higher levels of neutralizing antibodies (Figure 2). No relationship was evident between age and cross-neutralization of the GL8 pseudotype. It was also notable that many samples contained either weak or no neutralizing antibody activity, and occasionally antibodies were detected that appeared to enhance infection (Figure 2).

### 3.3. Broadly Neutralizing Antibodies

The neutralizing antibody response to HIV develops over time and there is considerable variation between patients in both the potency of the response and the time it takes to develop [42]. For example, Richman *et al*. found that while one patient achieved a neutralizing titer >1000 by 6 months post-infection, a second patient had no significant neutralizing antibody by 11 months post-infection [42]. The neutralizing response against homologous virus develops more slowly than the humoral response to Env *per se*, a pseudovirion-based assay detecting significant neutralizing antibodies by 30 weeks post-infection while anti-V3 peptide reactivity was evident as early as 10 weeks post-infection [44]. Comparisons of neutralizing activity in sera from HIV-1 infected individuals have indicated that the breadth of the neutralizing response varies between individuals. In many cases activity is narrow and strain-specific while sera from others may display broad intra-clade, and more rarely inter-clade, neutralizing antibody [45,47,55,56]. The sera that neutralize diverse HIV-1 isolates are deemed to contain broadly neutralizing antibodies and the induction of such broadly neutralizing antibodies is the ultimate goal of HIV vaccine design. Previous studies have suggested that true broadly neutralizing antibody are relatively rare and that much of the broad neutralizing activity within such sera is directed against gp120, targeting the CD4 binding site [57], although other specificities are thought to contribute [57,58]. The concept of broadly neutralizing antibodies has been extended further with the identification of “elite neutralizers”, a population of HIV-infected individuals (representing 1% of the 1,234 individuals screened) possessing serum antibodies capable of neutralizing more than one strain of virus within a clade and across at least four clades [47].

Little is known about the breadth of neutralizing activity within sera from FIV-infected cats. Therefore, we conducted a pilot survey of neutralizing activity in cats naturally infected with FIV, screening 345 plasma samples for neutralizing antibodies against the GL8 isolate. The samples containing neutralizing antibody were collected from cats aged between 18 months and 14 years. Subsequently, samples which strongly neutralized GL8 were tested against other isolates to assess the breadth of cross-reactivity. Of the 345 plasma samples that were tested, 30 (8.7%) strongly (*i.e.*, ≥80% at a 1:10 dilution) neutralized GL8 Env-bearing pseudotypes. As isolates within the UK are almost exclusively subtype A [59], the absence of anti-GL8 neutralizing antibodies in the majority of the samples may suggest that the majority of infections either elicit strain-specific responses or do not elicit strong neutralizing antibody responses. Alternatively, the GL8 isolate may represent a strain of FIV that is largely resistant to neutralization. The 30 plasma samples containing neutralizing antibodies that strongly neutralized GL8 were tested next for the ability to neutralize HIV(FIV) pseudotypes bearing Envs from the U.S.A. subtype A strain PPR and USA subtype C strain CPG-41. 6 samples were identified that neutralized GL8, PPR and CPG41 strongly; these samples were screened further against pseudotypes bearing Envs from a panel of 19 primary isolates, revealing two sera with broad cross-neutralizing activity, 206394 and 178639 (Table 1). Thus from an initial screening of 345 samples, only samples two exhibited broad, inter-subtype neutralizing activity, representing 10% of the plasma samples containing detectable neutralizing antibodies or 0.9% of all the samples tested from cats naturally infected with FIV. This proportion is similar to recent reports for HIV infections; plasma from 7 of 191 viremic patients (3.7%) and 3 of 174 aviremic patients receiving antiretrovirals (1.7%) contained broadly reactive neutralizing antibody [60].

### 3.4. Determinants of FIV Neutralization

The nature of the assay systems used to assess FIV neutralization *in vitro* indicated initially that the V3 loop was a major determinant for virus neutralization [32,33]. Subsequent studies revealed that the significance of antibodies targeting this region to the neutralizing response had been over-emphasized by the cellular substrate used to quantify neutralization. Formerly, assays were based on inhibiting infection of CrFK cells with “CrFK-adapted” strains of virus. The challenge viruses used in these assays were selected for growth in CrFK cells and, during this process of CrFK-adaptation, viruses were adapted to CD134-independent infection mediated by a direct interaction with CXCR4 (analogous to CD4-independent infection with HIV). As the V3 loop plays a critical role in the FIV Env-CXCR4 interaction, CrFK-based assays exaggerated the importance of neutralizing antibodies binding this region [61]. Accordingly, vaccines targeting selectively the FIV V3 loop proved ineffective at preventing infection [62]. A possible role for V3 in virus neutralization post-engagement of the viral receptor has been suggested [63] although neutralizing activity would only appear to be evident in assays performed *in vitro* in the presence of soluble forms of the viral receptor, CD134.

Assays employing IL2-dependent T cells have suggested that there is little correlation between the efficiency of virus neutralization and the response to V3. In contrast, several studies have indicated important roles for the V4 and V5 regions in virus neutralization [64–67]. A possible linear epitope in the V5 region of the 19K1 strain was investigated using synthetic peptides [68]. A single mutation at position 560 in V5 may be involved concurrently with an additional mutation at 483 in the V4 loop to form a conformation-dependent determinant for neutralizing antibody [68]. Independently, two mutations that resulted in the creation of potential sites for N-linked glycosylation in V4 (K481N) and V5 (S557N) contributed to the conversion of FIV from a neutralization-sensitive to neutralization-resistant phenotype [65,69]. Further evidence for the importance of the V5 region of FIV to the control of viral replication *in vivo* has been uncovered in analyses of the evolution of a molecular clone of the GL8 strain over time. Five years post-infection of cats with an infectious molecular clone of FIV, viral variants were isolated from PBMC, their nucleic acid sequences determined and their biological properties investigated. Variants had acquired mutations in the V5 loop that mediated escape from homologous neutralizing antibody [52].

**Figure 3.**
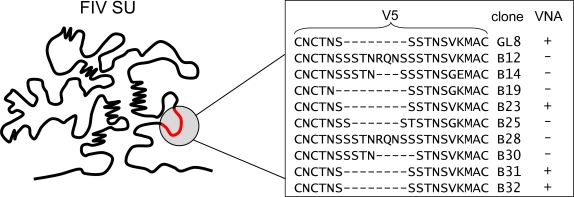
Amino acid sequence variation in the V5 loop of viral variants isolated from a GL8-infected cat. Five years post-infection with a molecular clone of GL8, variants were isolated that were either sensitive (+) or resistant (−) to virus neutralizing antibody. Variants that were sensitive to homologous VNA had identical V5 sequences. (Adapted from [52]).

By exchanging V5 loops between neutralization-resistant and neutralization-sensitive variants, the specificity of the neutralizing response was confirmed as targeting V5. The mechanism of escape from neutralization differed between variants, involving either shortening, lengthening or mutating of V5 (Figure 3). The importance of V5 in FIV neutralization is not restricted to subtype A viruses; in a separate study a neutralization-resistant Japanese subtype B strain of FIV (NG4) escaped neutralization by homologous and heterologous sera following the elimination of a potential site for N-linked glycosylation in V5 [35]. The V5 loop of FIV Env is highly variable among isolates, with variation occurring not only within the amino acid sequence but also in the length of the central region (up to 14 residues) towards the end of the loop structure formed by disulfide bond linkage. Length polymorphisms in this region of the FIV Env protein are attributable largely to the reiteration of codons encoding serine and threonine residues, residues that possess hydroxyl side chains and may, potentially, undergo O-linked glycosylation. It is generally believed that O-linked oligosaccharides have only minor effects on the formation of the glycan shield on HIV Env compared to N-linked oligosaccharides (due to their small molecular size compared with N-linked glycans). However, as O-linked oligosaccharides have diverse structures with no common carbohydrate core, it is difficult to predict how efficient they may be in shielding neutralization epitopes, especially in regions where there are multiple adjacent sites for O-linked glycosylation [70]. X-ray crystallographic studies on HIV-1 gp120 have demonstrated that glycosylation may affect significantly the conformational stability of Env as well as having indirect effects on more distant sequences along the secondary structure. This in turn would affect the accessibility of epitopes for interactions with neutralizing antibody [71]. Like FIV, HIV-1 evades the Env-targeted humoral immune response by the incorporation of amino acid substitutions, length polymorphisms (insertions and deletions) and by altering the pattern of glycosylation [42,72–76]. Recent data have indicated that population-level adaptation of HIV-1 gp120 to humoral immunity over time has been associated with an enhanced resistance of the virus to neutralizing antibody and that this resistance has coincided with both an increase in length of the variable loops and an increase in the number of potential sites for N-linked glycosylation [77]. It is striking that both the FIV and HIV Envs should incorporate similar changes in response to the host immune response and indicates that similar mechanisms are at play in FIV-infected cats as have been observed in HIV-infected individuals. By comparing and contrasting the two infections, valuable insights may be obtained into immunity to lentiviral infection and the prospects for the development of Env-based vaccines.

Escape from neutralizing antibody is facilitated by the acquisition of non-synonymous mutations over time and thus the rate of viral evolution in the infected animal will influence the ability of the virus to overcome a neutralizing response. Studies examining viral sequence variation over time in FIV infected cats have concluded that in comparison with other lentiviruses, FIV is remarkably stable genetically [51,78–80], similar to observations with bovine immunodeficiency virus (BIV) infection of cattle [81]. It possible that the low rates of FIV evolution reported to date reflect slower replication kinetics of FIV. Previous studies have demonstrated that the degree of SIV divergence following infection of macaques was closely linked to the rate of viral replication. Thus, a virus which replicated more slowly diverged from the ancestral strain by 0.6% after 75 weeks while a rapidly replicating strain diverged by 1.4% after 75 weeks [82]. If FIV has a reduced replication rate, this may impact upon the frequency at which mutations facilitating escape from neutralization arise in infected cats.

### 3.5. Neutralizing Antibodies Targeting the Membrane-Proximal External Region of FIV

Among the broadly neutralizing antibodies that have been identified as targeting HIV-1, three monoclonal antibodies (2F5, 4E10 and Z13) were found to recognize determinants on the viral transmembrane protein gp41. The antibodies bind to a highly conserved stretch of the transmembrane region of gp41 that lies immediately adjacent to the external surface of the viral lipid envelope, a region referred to as the membrane-proximal external region (MPER) (reviewed comprehensively in [83]). An analogous region has since been identified in gp41 of FIV and peptides derived from this region are potent inhibitors of viral entry [84,85]. When a synthetic peptide derived from the FIV MPER (peptide 59) was coupled to Qβ virus-like particles and used to elicit anti-MPER antibodies in cats, potent anti-peptide responses were detected by enzyme-linked immunosorbent assay (ELISA) [85]. However, when the sera were screened for binding to the MPER, in the context of whole FIV virions, neither virus binding nor virus neutralization were detected [85]. The failure of peptide 59 to induce neutralizing antibodies may have been due to the peptide not being presented in the correct context; the MPER of HIV-1 is in close apposition with the viral envelope and thus may only adopt the conformation associated with the induction of neutralizing antibodies when in association with a lipid membrane. In an attempt to mimic the context in which the FIV MPER is presented on intact virions, cats were immunized with a lipoylated analogue of peptide 59 (lipo-P59 [86]). Immunization with lipo-P59 induced antibodies that were detectable by ELISA and which bound to FIV virions, in contrast to previous findings with the non-lipoylated form of the same peptide [87]. However, in comparison with sera from FIV-infected cats, the sera from lipo-P59-immunized cats contained no neutralizing antibodies [87], suggesting that although the lipoylation of the peptide had altered its conformation and immunogenicity, it did not offer a viable approach to the development of an FIV vaccine. Intriguingly, using a T cell based neutralization assay based on MBM cells (an IL2-dependent cell line similar to MYA-1 cells [88]), the sera from the immunized cats were found to enhance FIV infection, an effect that could be adsorbed by pre-incubation with lipo-P59 [87]. While disappointing from a vaccine design perspective, these findings may prove informative in interpreting why some FIV vaccines that failed to induce protective immunity, appeared to enhance infection following challenge [62,69,89–93].

### 3.6. Induction of FIV Neutralizing Antibodies by Vaccination

Protection against FIV infection was first demonstrated by Yamamoto and colleagues using whole inactivated virus and cells prepared from the FL4 cell line, an IL-2 independent cell line persistently infected with the subtype A Petaluma strain of FIV (PET) [94,95]. However, immunity induced by the FL4-based vaccine did not extend to heterologous subtype A strain GL8 [96]. Further, protection afforded by the FL4 vaccine did not correlate with the potency of the neutralizing antibody response when assessed on a CrFK-based (V3-biased) assay and could not be replicated using affinity-purified Env [97]. It was noted that there were qualitative and quantitative differences in the nature of the immune response induced by the FL4-based vaccine and an affinity-purified Env-based vaccine [97]. Moreover, the FL4-based vaccine induced both antibodies that reacted with host cell proteins [97] and FIV-specific cytotoxic T cells [98,99], suggesting that additional factors may have contributed to immunity, a finding underlined by the observation that DNA vaccines could induce protection from FIV PET infection in the absence of a detectable humoral immune response [39]. While passive transfer experiments indicated that humoral immunity could be transferred to kittens from cats, the transfer experiments were followed by challenge with the neutralization-sensitive FIV PET strain of FIV [100].

Why do FL4-based vaccines fail to protect against heterologous strains of virus? One possibility is that the prototypic vaccines failed to induce neutralizing antibody against conformational epitopes that would be required to confer heterologous immunity; evidence to date would suggest that the majority of the neutralizing responses that develop *in vivo* are strain-specific and target V4 and V5 [35,52,65–69,101] while broadly neutralizing antibodies are rare. The breadth of the immunity induced by FL4-based vaccines has been extended by the introduction of heterologous strains of virus into the FL4 cell line [102–104], and such vaccines have afforded limited success. However, whether the heterologous immunity induced by such vaccines reflects a qualitative or quantitative improvement in the neutralizing antibody response remains to be established. Broad-spectrum protection has yet to be achieved. It is possible that some strains of FIV are inherently more resistant to neutralizing antibody and that, as FIV has become adapted to its feline host, the virus has enhanced its ability to evade humoral immunity by shrouding itself in N-linked glycans extending its variable loops, similar to the mechanism that has been postulated for HIV-1 [77]. If this is the case, then it is possible that retargeting the immune response by selective deglycosylation of immunogens may reveal novel cryptic epitopes that may induce neutralizing antibodies and offer hope for the development of more highly efficacious FIV vaccines.

## Figures and Tables

**Table 1.** Broad neutralization of FIV by plasmas from two FIV-infected cats. Percentage neutralization of HIV (FIV) pseudotypes at a plasma dilution of 1:10 was calculated relative to a “no plasma” control sample.

			**Plasma**	

**Virus**	**Origin**	**Subtype**	**178639**	**206394**
GL8	UK	A	99	95
180638	UK	A	99	90
171838	UK	A	99	99
180260	UK	A	96	96
180140	UK	A	90	97
178721	UK	A	99	97
179200	UK	A	83	97
206394	UK	A	83	93
0425	UK	A	99	96
0827	UK	A	99	95
1419	UK	A	95	96
PPR	USA	A	98	99
B2542	USA	B	99	99
KNG2	Japan	B	96	99
TM2	Japan	B	99	99
Pisa M2	Italy	B	93	100
Leviano	Brasil	B	90	100
CPG41	USA	C	96	99
Poose	Sri Lanka	-	73	100
LLV-B	Tanzania	-	51	16
